# Design and Harmonization Approach for the Multi-Institutional Neurocognitive Discovery Study (MINDS) of Adult Congenital Heart Disease (ACHD) Neuroimaging Ancillary Study: A Technical Note

**DOI:** 10.3390/jcdd10090381

**Published:** 2023-09-06

**Authors:** Ashok Panigrahy, Vanessa Schmithorst, Rafael Ceschin, Vince Lee, Nancy Beluk, Julia Wallace, Olivia Wheaton, Thomas Chenevert, Deqiang Qiu, James N Lee, Andrew Nencka, Borjan Gagoski, Jeffrey I. Berman, Weihong Yuan, Christopher Macgowan, James Coatsworth, Lazar Fleysher, Christopher Cannistraci, Lynn A. Sleeper, Arvind Hoskoppal, Candice Silversides, Rupa Radhakrishnan, Larry Markham, John F. Rhodes, Lauryn M. Dugan, Nicole Brown, Peter Ermis, Stephanie Fuller, Timothy Brett Cotts, Fred Henry Rodriguez, Ian Lindsay, Sue Beers, Howard Aizenstein, David C. Bellinger, Jane W. Newburger, Laura Glass Umfleet, Scott Cohen, Ali Zaidi, Michelle Gurvitz

**Affiliations:** 1 Department of Radiology, UPMC Children’s Hospital of Pittsburgh, 4401 Penn Ave. Floor 2, Pittsburgh, PA 15224, USA; vanessa.schmithorst@chp.edu (V.S.); rcc10@pitt.edu (R.C.); vkl2@pitt.edu (V.L.); beluknh@upmc.edu (N.B.); julia.wallace@chp.edu (J.W.); hoskoppalak@upmc.edu (A.H.); 2 Department of Pediatric Radiology, Children’s Hospital of Pittsburgh of UPMC, 45th Str., Penn Ave., Pittsburgh, PA 15201, USA; 3 HealthCore Inc., 480 Pleasant Str., Watertown, MA 02472, USA; olivia.wheaton@carelon.com; 4 Department of Radiology, Michigan Medicine University of Michigan, 1500 E Medical Center Dr., Ann Arbor, MI 48109, USA; tlchenev@med.umich.edu; 5 Congenital Heart Center, C. S. Mott Children’s Hospital, 1540 E Hospital Dr., Ann Arbor, MI 48109, USA; 6 Department of Radiology and Imaging Sciences, Emory School of Medicine, 1364 Clifton Rd., Atlanta, GA 30322, USA; deqiang.qiu@emory.edu; 7 Department of Radiology, The University of Utah, 50 2030 E, Salt Lake City, UT 84112, USA; james.lee@hsc.utah.edu; 8 Department of Radiology, Medical College of Wisconsin, 9200 W Wisconsin Ave., Milwaukee, WI 53226, USA; anencka@mcw.edu; 9 Department of Radiology, Boston Children’s Hospital, 300 Longwood Ave., Boston, MA 02115, USA; borjan.gagoski@childrens.harvard.edu; 10 Department of Radiology, Children’s Hospital of Philadelphia, 3401 Civic Center Blvd., Philadelphia, PA 19104, USA; bermanj@email.chop.edu; 11 Department of Radiology, Cincinnati Children’s Hospital Medical Center, 3333 Burnet Ave., Cincinnati, OH 45229, USA; weihong.yuan@cchmc.org; 12 Department of Radiology, University of Cincinnati College of Medicine, 3230 Eden Ave., Cincinnati, OH 45267, USA; 13 Department of Medical Biophysics, University of Toronto, 101 College Str. Suite 15-701, Toronto, ON M5G 1L7, Canada; christopher.macgowan@sickkids.ca; 14 The Hospital for Sick Children Division of Translational Medicine, 555 University Ave., Toronto, ON M5G 1X8, Canada; 15 Department of Radiology, Medical University of South Carolina, 171 Ashley Ave., Room 372, Charleston, SC 29425, USA; coatswor@msc.edu; 16 BioMedical Engineering and Imaging Institute, Icahn School of Medicine at Mount Sinai, 1470 Madison Ave., New York, NY 10029, USA; lazar.fleysher@mountsinai.org (L.F.); christopher.cannistraci@mssm.edu (C.C.); ali.zaidi@mountsinai.org (A.Z.); 17 Department of Cardiology, Boston Children’s Hospital, 300 Longwood Ave., Boston, MA 02115, USA; lynn.sleeper@cardio.chboston.org (L.A.S.); jane.newburger@cardio.chboston.org (J.W.N.); michelle.gurvitz@cardio.chboston.org (M.G.); 18 Department of Cardiology, University of Toronto, C. David Naylor Building, 6 Queen’s Park Crescent West, Third Floor, Toronto, ON M5S 3H2, Canada; candice.silversides@uhn.ca; 19 Department of Radiology and Imaging Sciences, Indiana University School of Medicine, 550 University Blvd., Indianapolis, IN 46202, USA; rupa.rad@gmail.com; 20 Department of Cardiology, University of Indiana School of Medicine, 545 Barnhill Dr., Indianapolis, IN 46202, USA; lwmarkha@iu.edu; 21 Department of Cardiology, Medical University of South Carolina, 96 Jonathan Lucas Str. Ste. 601, MSC 617, Charleston, SC 29425, USA; rhodesjf@musc.edu; 22 Department of Cardiology, Cincinnati Children’s Hospital Medical Center, 3333 Burnet Ave., Cincinnati, OH 45229, USA; lauryn.dugan@cchmc.org (L.M.D.); nicole.brown@cchmc.org (N.B.); 23 Department of Radiology, Texas Children’s Hospital, Houston, TX 77030, USA; prermis@texaschildrens.org (P.E.); fullers@chop.edu (S.F.); 24 Departments of Internal Medicine and Pediatrics, Michigan Medicine University of Michigan, 1500 E Medical Center Dr., Ann Arbor, MI 48109, USA; cottstim@med.umich.edu; 25 Department of Cardiology, Emory School of Medicine, 100 Woodruff Circle, Atlanta, GA 30322, USA; fred.rodriguez@emory.edu; 26 Department of Cardiology, The University of Utah, 95 S 2000 E, Salt Lake City, UT 84112, USA; ian.lindsay@hsc.utah.edu; 27 Department of Psychiatry, University of Pittsburgh School of Medicine, 3811 O’Hara Str., Pittsburgh, PA 15213, USA; beerssr@upmc.edu (S.B.); aizensteinhj@upmc.edu (H.A.); 28 Cardiac Neurodevelopmental Program, Boston Children’s Hospital, 300 Longwood Ave., Boston, MA 02115, USA; david.bellinger@childrens.harvard.edu; 29 Department of Neuropsychology, Medical College of Wisconsin, 9200 W Wisconsin Ave., Milwaukee, WI 53226, USA; lumfleett@mcw.edu; 30 Heart and Vascular Center, Medical College of Wisconsin, 8701 Watertown Plank Rd., Milwaukee, WI 53226, USA; scohen@childrenswi.org

**Keywords:** adult congenital heart disease, cognitive reserve, harmonization, multi-center neuroimaging, phantoms

## Abstract

Dramatic advances in the management of congenital heart disease (CHD) have improved survival to adulthood from less than 10% in the 1960s to over 90% in the current era, such that adult CHD (ACHD) patients now outnumber their pediatric counterparts. ACHD patients demonstrate domain-specific neurocognitive deficits associated with reduced quality of life that include deficits in educational attainment and social interaction. Our hypothesis is that ACHD patients exhibit vascular brain injury and structural/physiological brain alterations that are predictive of specific neurocognitive deficits modified by behavioral and environmental enrichment proxies of cognitive reserve (e.g., level of education and lifestyle/social habits). This technical note describes an ancillary study to the National Heart, Lung, and Blood Institute (NHLBI)-funded Pediatric Heart Network (PHN) “Multi-Institutional Neurocognitive Discovery Study (MINDS) in Adult Congenital Heart Disease (ACHD)”. Leveraging clinical, neuropsychological, and biospecimen data from the parent study, our study will provide structural–physiological correlates of neurocognitive outcomes, representing the first multi-center neuroimaging initiative to be performed in ACHD patients. Limitations of the study include recruitment challenges inherent to an ancillary study, implantable cardiac devices, and harmonization of neuroimaging biomarkers. Results from this research will help shape the care of ACHD patients and further our understanding of the interplay between brain injury and cognitive reserve.

## 1. Introduction

**Figure 1:**
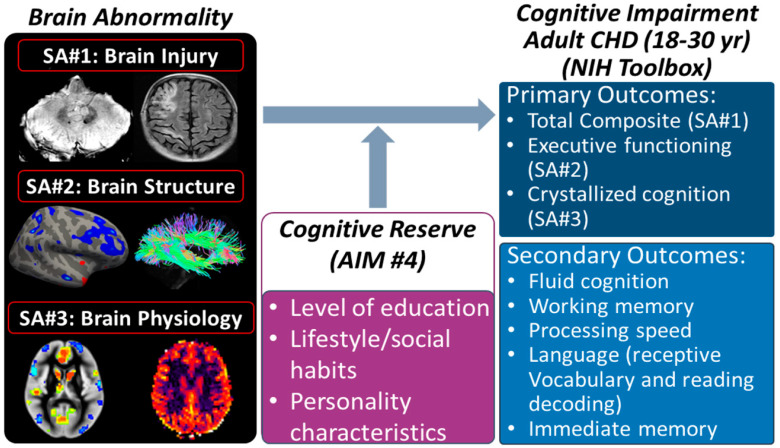
OVERVIEW/PREMISE: Neurocognitive impairment in CHD arises from a variety of etiologies, including not only brain injury but also structural and vascular abnormalities (**left**), which reflect deficits in a variety of neurocognitive domains (**right**). These deficits may be modified by cognitive reserve (CR) (**center**) in a similar manner as in aging populations and younger TBI populations. CR is associated with “enrichment” due to factors such as education, occupation, personality, etc.

Dramatic advances in the management of congenital heart disease (CHD) have improved survival to adulthood from less than 10% in the 1960s to over 90% in the current era [1]. With this shifting demographic, adult CHD (ACHD) patients now outnumber pediatric CHD patients [2]. Improved survival, however, has exposed that adults with CHD demonstrate domain-specific neurocognitive deficits, such as impairment in executive function, which is associated with reduced quality of life, lower educational attainment, and less social interaction [3,4,5,6,7,8]. These deficits are associated with cumulative risk factors across the lifespan, ranging from fetal risk factors (pathogenic genetic variants affecting both brain and heart development and reduced cerebral oxygen delivery) to postnatal perioperative hemodynamic instability and other medical risk factors in childhood to adult-onset atherosclerotic cerebrovascular disease [9,10]. Later in the lifespan, ACHD patients may be at risk for early dementia [11]. Acquired brain injury and structural/physiological alterations (i.e., reduced cortical thickness, brain connectivity, regional cerebral blood flow) in the immature CHD brain have been shown to correlate with neurocognitive deficits throughout the adolescent period. Neurocognitive deficits in both pediatric and adult subjects may also be related to cognitive reserve (CR) [12,13,14,15,16,17,18,19,20,21,22,23], a protective factor preventing or reducing cognitive impairment in response to a given insult, possibly via differential recruitment of brain structures or brain networks (Figure 1). The relationship between brain abnormalities in young ACHD patients, specific neurocognitive deficits, and important proxies of CR are poorly understood knowledge gaps that are addressed by this study.

Here, we present an ancillary study to the National Heart, Lung, and Blood Institute (NHLBI)-funded Pediatric Heart Network’s (PHN) “Multi-Institutional Neurocognitive Discovery Study (MINDS) in Adult Congenital Heart Disease (ACHD)”. The parent MINDS–ACHD study will recruit 500 participants with moderately or severely complex CHD aged 18–30 years to examine their objective and subjective neurocognitive functioning and identify genetic predictors of neurocognitive dysfunction [24]. We will quantitate multimodal neuroimaging biomarkers (e.g., brain injury, structure, and physiology) obtained from Magnetic Resonance Imaging (MRI) and examine their association with specific cognitive deficits in the ACHD population [25,26,27,28,29,30]. Whereas similar neuroimaging studies have characterized large-scale populations such as the Alzheimer’s Disease Neuroimaging Initiatives (ADNI), few have been performed in the ACHD population [31,32,33,34,35,36]. We will leverage the MINDS–ACHD parent study data, including the NIH Toolbox neuropsychological battery/clinical data/biological samples. Additionally, we will apply our established neuroimaging harmonization, which we currently use for the PHN Single Ventricle Reconstruction (SVRIII) multi-center brain connectome study (R01-HL128818; PI-Panigrahy), to measure neuroimaging biomarkers in ACHD patients at the same PHN sites.

Our overarching hypothesis is that adults with CHD exhibit vascular brain injury and structural/physiological brain alterations that are predictive of specific neurocognitive deficits. These changes may be influenced by behavioral and environmental enrichment proxies of CR (e.g., level of education and lifestyle/social habits). The aims of this study are as follows: Aim #1 (brain injury): To determine if vascular-related brain injury (cortical infarcts, hemosiderin lesions, and white matter hyperintensity) is associated with specific neurocognitive deficits using the NIH Toolbox total composite score; Aim #2 (brain structure): To determine if reduced fronto-temporal cortical thickness and white matter connectivity are associated with specific neurocognitive deficits using the NIH Toolbox frontal executive sub-score; Aim #3 (brain physiology): To determine if reduced cerebrovascular reserve (regional cerebral blood flow/resting BOLD imaging) is associated with specific neurocognitive deficits using the NIH Toolbox crystallized composite score; Aim #4 (cognitive reserve): To determine if the associations between neuroimaging biomarkers and neurocognitive outcomes in ACHD patients are modified by behavioral and environmental enrichment proxies of CR using traditional statistical models and machine learning techniques.

## 2. Methods and Designs

### 2.1. Introduction to Parent and Ancillary Study

The Multi-Institutional Neurocognitive Discovery Study (MINDS) in Adult Congenital Heart Disease (ACHD) is a multi-center cross-sectional analysis of the neurocognitive function of those with moderately or severely complex CHD, ages 18–30 [24]. This study will enroll 500 participants in 4 groups of 125 individuals, each with d-transposition of the great arteries, tetralogy of Fallot, a single ventricle, and other moderately or severely complex CHD lesions [24]. The primary aim of the parent study is to examine objective and subjective neurocognitive functioning in adults with CHD [24]. The secondary aims of the parent study are (1) to determine the concurrent association of objective and subjective neurocognitive deficits with demographic and clinical factors and (2) to identify genetic predictors of neurocognitive outcomes [24]. The proposed MINDS neuroimaging ancillary study (n = 156) will leverage the neurocognitive testing (NIH Toolbox) and clinical/demographic risk factor data collected as part of the parent MINDS study. Participants in the MINDS study who also participate in the proposed ancillary study will be asked to undergo one brain MRI (without contrast) and complete a questionnaire related to various potential proxies of cognitive reserve (CR) such as education, personality, occupation, and lifestyle/social habits.

### 2.2. Enrollment for MINDS Ancillary Neuroimaging Study

Entry criteria for the ancillary neuroimaging study require participation in the parent MINDS study. Exclusion criteria included pregnancy, claustrophobia, orthodontic braces, pacemakers, and other implantable metal devices that are not MR-compatible at 3 T. For this ancillary study, we conservatively assume a 25% consent rate among those enrolled in MINDS. We are expecting approximately 15% of data points to be potentially unanalyzable due to motion artifacts or technical issues. We anticipate enrolling 156 participants at 13 sites (an average of 12 participants per site). Both sexes will be recruited for this study. To determine the neuroimaging capabilities at the MINDS clinical centers, a detailed MRI questionnaire was completed by each site for information about the capabilities of 3 T MRI scanners. We also queried about the availability and interest of neuro-based personnel, including the neuroradiologist, MR physicist, and MR technologist’s presence/capability.

### 2.3. Neurocognitive Measures—NIH Toolbox (From the Parent MINDS Study) and Questionnaire (New in the Ancillary Study)

The parent MINDS study will utilize the NIH Toolbox Cognition Battery, a brief, computerized assessment of cognition [37]. The battery has seven subtests assessing working memory, processing speed, executive functioning (inhibitory control and cognitive flexibility), language (receptive vocabulary and reading decoding), and immediate memory. It generates three composite scores: overall, crystallized, and fluid cognition. Each composite score and subtest has demonstrated robust developmental effects and expectable correlations with “gold standard” tests of similar abilities [38]. The NIH Toolbox has now been used in multiple clinical neurological adult populations, including traumatic brain injury, stroke, spinal cord injury, and prodromal dementia [39,40,41]. Our ancillary study will add a questionnaire to assess proxies of cognitive reserve. We utilized our cognitive neuroscience and neuropsychology expertise to develop the CR questionnaire tailored to our young ACHD population. While CR is a widely used concept in elderly populations involving studies of normal aging [42,43] as well as Alzheimer’s disease [42,44] and other dementias [45], CR has also been used in younger adults and children with traumatic brain injury [46,47,48,49]. CR is known to correlate with lifestyle and cognitive factors, such as level of education, type of occupation, vocabulary, and frequency of reading, which are frequently referred to as “cognitive enrichment” or “intellectual enrichment” factors [42,43]. Exploratory measures (e.g., Mini-Mental Status Exam (MMSE) and idea density) will also be obtained [50,51,52,53].

### 2.4. MR Imaging Protocols and Pulse Sequences

We harmonized select MRI pulse sequences, including the 3D-T1 (anatomic-macrostructure), diffusion (microstructure), and resting BOLD (functional) sequences, following previously implemented guidelines by the multi-vendor ABCD study [54]. The MRI protocol also included additional sequences to evaluate for acquired brain injury, including SWI (hemosiderin/microbleed deposition) and FLAIR 3D isotropic 1 mm imaging (white matter disease/lesions/hyperintensity and cortical infarcts) (Supplemental Table S1A,B).

### 2.5. Specific Aim 1: Vascular-Related Brain Injury—Is It Associated with Neurocognitive Deficits?

#### 2.5.1. Rationale

Vascular-related acquired brain injury is common in aging adult non-CHD patients and is characterized by three hallmark lesions [55]: (1) cortical infarcts; (2) hemosiderin deposition; and (3) white matter hyperintensities (WMH). Importantly, these lesions have been detected in immature and adolescent brains in small studies of adults with CHD [56]. Studies in elderly non-CHD patients with small vessel ischemic disease (as identified by WMH) confirm that higher CR (i.e., educational attainment) attenuates the negative impact of WMH on cognitive function [57]. These lesions have been identified in four CHD cohorts: one infant (biventricular CHD) [58] and three separate adolescent CHD cohorts (TGA [59], ToF [60], and Fontan [61]). The regional pattern of microbleeds has been well documented in both the aging [62,63,64,65] and non-CHD dementia populations and is predictive of cognitive dysfunction [62,63,64,65,66,67,68,69].

#### 2.5.2. Analysis Plan

The primary exposure (predictor) is the binary presence/absence and/or number/volume of brain lesions. The primary outcome is the NIH Toolbox total composite score, and secondary outcomes are composite sub-scores (crystalized and fluid) and domain-specific (i.e., executive function) sub-scores. Secondary exposure will be composite brain injury scores and lesion subtypes derived from a combination of observation and quantitation. We will measure the agreement of brain injury scores calculated by two independent neuroradiologists, and all disagreements will be adjudicated. To determine if qualitative and quantitative metrics of brain injury predict worse neurocognitive outcomes (composite scores—NIH Toolbox) in ACHD patients, we use linear regression and will regress each neurocognitive test composite score on each of the brain injury metric variables. A normalizing or variance-stabilizing transformation will be applied to the outcome scores if necessary to meet model assumptions. Bootstrapping/resampling methods will be employed to assess the reliability of parameter estimates from both the univariate and multivariable models that are constructed. The brain injury metrics will also be modeled as nonlinear terms (e.g., categorical and other transformations) if exploratory nonparametric modeling indicates that the association is not linear. Sensitivity analyses will also be conducted to control for the time between brain MRI and neurocognitive testing. Sex as a biological variable, cardiac subtypes, and other clinical factors collected from the parent MINDS study will be explored as both covariates and effect modifiers. To account for multiple comparisons, a false discovery rate (FDR) correction will be used; results will be deemed significant at an FDR corrected q < 0.05.

#### 2.5.3. Aim 1 Power Analysis

The primary predictor, which is the presence/absence of brain injury lesions, is estimated to have a 20% prevalence. Therefore, with our target sample size of 156 patients, group sizes will be 39 and 117. Our past research suggests that the expected difference in neurocognitive outcomes between lesion and no-lesion groups is greater than 2 SDs [61]. Therefore, with 156 patients, for a two-sided 0.05 level test, there is 85% power to detect a minimum clinically important difference (0.56 SDs) in the mean NIH composite scores of the lesion and no-lesion groups and higher power to detect larger differences that have been observed in related research. (For the secondary predictor of hemosiderin lesions, while the detectable difference between the two groups will be smaller [61], the expected prevalence is 30%.)

### 2.6. Specific Aim 2: Brain Structure—Is It Associated with Neurocognitive Deficits?

#### 2.6.1. Justification

Cortical thickness and diffusion tensor imaging have been used to (1) demonstrate structural fronto-temporal abnormalities in the immature and adolescent CHD brain by our groups [70,71,72,73,74,75,76,77,78] and (2) represent neural correlates of cognitive reserve (CR) in non-CHD aging and dementia cohorts [25].

#### 2.6.2. Interpretation

These data suggest the structural vulnerability of the prefrontal region and the default mode network (DMN) in adolescent CHD, a large-scale brain cognitive network that is metabolically active at rest. In support of this premise, a recent small neuroimaging study of 10 adult cyanotic CHD patients (mean age ~40 years) demonstrated lower cortical thickness in the anterior and posterior DMN and correlation with serum biomarkers of neuroinflammation and endothelial dysfunction, which are also hallmarks of energy failure [79].

#### 2.6.3. Methodology

See Supplemental Methods and Supplemental Figures S1 and S2.

#### 2.6.4. Aim 2 Analysis Plan and Brain–Outcome Relationship

The primary exposure (predictor) is the cortical thickness of the prefrontal region and fractional anisotropy/GFA of the superior longitudinal fasciculus (continuous measurements), as this tract is important for both executive and language functions. The primary outcome is the executive function subscore of the NIH Toolbox, and the secondary outcomes will be the composite scores and domain-specific sub-scores. Secondary predictors are cortical thickness and white matter connectivity measures of other regions of the brain. These measures will be examined for their association with the secondary outcomes of processing speed, memory, and language sub-scores from the NIH Toolbox. To determine if structural metrics predict poor neurocognitive outcomes in young adult CHD patients, we will use linear regression and regress each neurocognitive test sub-score on prefrontal cortical thickness and FA of SLF from DTI tractography. A normalizing or variance-stabilizing transformation will be applied to the outcome score, if necessary, to meet model assumptions. The structural imaging metrics will also be modeled as nonlinear terms (e.g., categorical and other transformations) if exploratory nonparametric modeling indicates that the association is not linear. FDR correction will be used to correct for multiple comparisons, and results will be deemed significant at FDR corrected q < 0.05. If the number of structural metrics to evaluate becomes large, we will employ techniques that control for multiplicity in testing, such as the Simes–Hochberg method. Sex as a biological variable, cardiac subtypes, acquired brain injury from Aim #1, and other clinical factors collected from the parent MINDS study will be explored as both covariates and effect modifiers.

#### 2.6.5. Aim 2 Power Analysis

With 156 patients and a two-sided 0.05 level test of whether the correlation between NIH sub-score and cortical thickness or white matter measurement differs from zero, we will have 85% power to detect correlations |R| of 0.24 or larger and 95% power to detect correlations of |R| = 0.28 or larger. Our recent research in adolescents with CHD has found that correlations between DTI/white matter connectivity and cognitive measures are of the magnitude of |R| = 0.6 or larger [80]. Therefore, with 156 patients, we have high power to detect correlations of the magnitude expected for the proposed study population.

### 2.7. Specific Aim 3: Brain Physiology—Is It Associated with Neurocognitive Deficits?

#### 2.7.1. Rationale

Cerebrovascular reserve or reactivity (CVR) refers to the capacity for increased cerebral blood flow (CBF) over baseline or “resting state” [81,82]. Impaired CVR has been implicated in adverse outcomes in many pathologies, including stroke [83], cerebrovascular disease such as atherosclerosis [84], anemia [85], mild cognitive impairment [86], and Alzheimer’s disease [87]. Mechanistically, the vasculature of the brain is unable to respond to increased metabolic demand, whether resulting from cognitively related neuronal activity or from an insult (whether vascular, neuronal, or synaptic in nature). CHD patients are known to have vascular abnormalities throughout their lifespan, including impaired autoregulation in infancy [88], that are associated with neurodevelopmental deficits [89]. Older CHD patients are at greater risk for acquired cardiovascular co-morbidities, resulting in an increased scale of neurovascular disease [9], a high risk of stroke [90,91,92], and microvascular ischemic disease. These considerations led us to hypothesize that impaired CVR may be a key factor underlying adverse neurocognitive outcomes in adult CHD patients. Typical methods for estimating CVR involve a vasoactive challenge, either an acetazolamide challenge [93,94] or a hypercapnic challenge induced via exogenous CO_2_ administration [82] or via breath-holding [95]. Then, either arterial-spin labeling (ASL) is used to directly estimate CBF or a BOLD signal is used as a proxy (as BOLD contrast is highly correlated with CBF). However, a vasoactive challenge is unsuitable for our ACHD population due to the prevalence of cardiorespiratory risk factors in this population. Therefore, we propose to use two methods available for the estimation of CBF without a vasoactive challenge. A previously validated method [96] has shown that if the MRI acquisition rate is sufficiently fast (TR ~400 ms), CVR may be estimated via resting-state BOLD acquisition as end-tidal CO2 (PETCO2) may be directly estimated from the global BOLD signal [97] and the BOLD time course correlated with it, which yields superior results compared to direct measurement of PETCO2 [97,98]. These TRs are readily obtainable even for whole-brain acquisitions via simultaneous multi-slice (SMS) MRI acquisition techniques [99]. As such, we will combine the ASL (cerebral blood flow-CBF measures) and BOLD imaging to develop a non-invasive MRI proxy of neurovascular function (pnvf), defined as the capability of the vasculature to respond to the baseline metabolic demand (e.g., as compared to a hypothetical “ground state” with zero neuronal activity). While pnvf is not identical to CVR and may be closer to a proxy of neurovascular function, it is relevant since resting-state activity also underlies cognitive function. A brain architecture that handles the task of meeting resting-state metabolic and connectivity demands more efficiently will likewise handle the task of meeting the additional demands posed by cognitive activity more efficiently. We will use functional connectivity strength (FCS) as a proxy for baseline metabolic demand, as functional connectivity and CMRO2 have been found to be tightly correlated, with certain regions of the brain forming a densely interconnected “rich club” with high metabolic demand [100,101,102]. Additionally, not only metabolism but also regional FCS increases relative to baseline in response to increased cognitive demand [103], indicating a “connectivity demand” for cognitive activity associated with metabolic demand. Thus, we can use the CBF/FCS ratio as a proxy for neurovascular function (as this ratio indicates how well the vasculature is supplying for resting-state metabolic demand), and it has been shown to be altered in neuropsychiatric disorders such as schizophrenia [104] as well as decrease with healthy aging [105]. Since CBF and FCS are both positive definite, we will instead compute the negative of the FCS/CBF ratio due to signal-to-noise ratio (SNR) considerations without impacting the direction of any detected relationships.

#### 2.7.2. Methodology

See Supplemental Methods and Supplemental Figure S3.

#### 2.7.3. Aim 3 Analysis Plan

Our primary analysis is to determine if physiological metrics predict poor neurocognitive outcomes (primary outcomes will be Crystallized and Fluid Composite, and secondary outcomes will be other domain sub-scores of the NIH Toolbox) in young ACHD patients. We will use linear regression and regress each neurocognitive test composite score on the negative FCS/CBF ratio (anterior default mode) and other physiological metrics (subcortical region and peri-Sylvian/salience network). As a secondary analysis, we will also regress FCS and CBF separately on NIH toolbox scores. Sex as a biological variable, cardiac subtypes, and other clinical factors collected from the parent MINDS study will be explored as both covariates and effect modifiers.

GLM voxel-based analyses: Each metric will be correlated with neurocognitive outcome on a voxel-wise basis, with age and sex as adjusted covariates. Results will be deemed significant at an FDR-corrected q < 0.05.

#### 2.7.4. Aim 3 Power Analysis

With 156 patients and a two-sided 0.05 level test of whether the correlation between NIH Toolbox cognitive scores and CVR differs from zero, we will have 85% power to detect correlations with |R| values of 0.24 or larger. Our estimate of the correlation is between 0.25 and 0.3, dependent on the brain network region [DMN (default mode network)/CEN (central executive network)], yielding sufficient power.

### 2.8. Specific Aim 4: Cognitive Reserve—Does It Modify Associations between Imaging Biomarkers and Cognitive Outcome?

#### 2.8.1. Rationale

**Figure 2:**
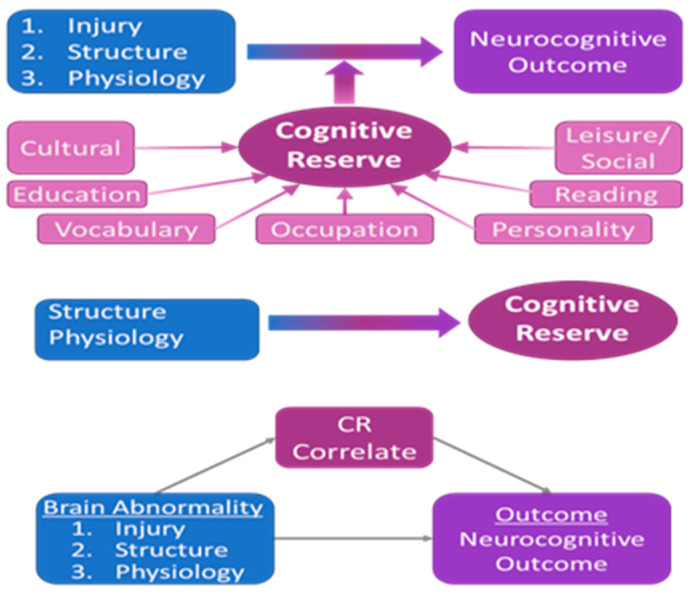
Cognitive Reserve modeling Top: Modeling and optimization of CR as a latent variable, a combination of behavioral, educational, social, and personality measures; Middle: Correlation structure/physiology yields CR correlates; Bottom: A mediation model tests whether CR correlates are markers of brain plasticity.

CR is a widely used concept in elderly populations, involving studies of normal aging [42,43] as well as neuropathologies such as Alzheimer’s disease [42,44] and other dementias [45]. It has also been used in younger adults and children with traumatic brain injuries [46,47,48,49]. CR is a factor that prevents or reduces functional impairment in response to neurological insults. As a research question, CR may be considered in two aspects: (1) How can it be accurately estimated [106] from behavioral, lifestyle, and environmental data (which often goes under the heading “cognitive enrichment” or “intellectual enrichment”), and (2) What are its structural and physiological correlates [21,107,108] (e.g., increased cortical thickness or brain size, reorganization of functional networks, altered white matter organization, etc.)? In elderly individuals, CR may be estimated via a combination of factors, including a measure of verbal ability related to crystallized intelligence (e.g., Wide Range Achievement Test, Peabody Picture Vocabulary Test, etc.), occupational complexity, leisure and social activities, personality traits, level of education, and level of parental education. These factors can moderate the relationship between disease burden and outcome [107,108,109,110,111,112,113]. (We note that it is also possible to model CR in a completely healthy population via an investigation of task-related functional activity [23]). Machine learning techniques [106] (e.g., random forest, neural network) may be used to find the optimal combination of factors by modeling CR as a latent variable (Figure 2, top). Then, once a suitable proxy of CR has been determined, one may investigate its neurophysiological underpinnings, better understand the mechanism underlying CR, and design appropriate interventions and preventative strategies, as has been performed for Alzheimer’s disease and other dementia patients [43,114]. Part of our overarching hypothesis is that CR operates in young adult CHD patients in a manner similar (but not identical) to that in elderly individuals and young adult/pediatric TBI patients, moderating the relationship between disease burden and clinical presentation. However, it is not known what factors may contribute to CR in this population, which may differ from the elderly and TBI populations, as this would be the first study investigating CR in CHD patients. We have identified three major contributors to the disease burden in CHD: injury, structure, and physiology. What are the factors contributing to CR in each of these contributors? It may also not be identical, as different aspects of CR may be necessary for each. Thus, for the same clinical presentation, there may be not only different structural and physiological etiologies but also different factors moderating the relationship. Our main goal for this aim is to elucidate these differences (question 1 of Aim 4), which are necessary before investigating their neurophysiological underpinnings (question 2). This information will constitute important data not only for the improved design of intervention strategies but also for future investigations of dementia risk.

#### 2.8.2. Methodology

Participants will be given a detailed questionnaire regarding occupation, level of education, level of parent education, leisure/cultural activities, personality (conscientiousness), reading frequency, food insecurities, socioeconomic status, and social activities. It contains 101 questions derived from previously utilized surveys [see below a–d] to assess the above-mentioned parameters. The participant will rate their response on a 1–5 scale, with 1 being “strongly disagree” and 5 being “strongly agree”. Since some participants may be as young as 18 years of age, enrollment in a university will be used as a surrogate measure of education. The NIH Toolbox Picture Vocabulary subtest (part of the Crystallized Cognition composite) and Word Reading measure will be used as the measures for vocabulary. Participants will also be asked to complete the Behavior Rating Inventory of Executive Function—Adult Version (BRIEF-A) self-report form. This includes 75 questions within nine nonoverlapping theoretically and empirically derived clinical scales: Inhibit, Self-Monitor, Plan/Organize, Shift, Initiate, Task Monitor, Emotional Control, Working Memory, and Organization of Materials for the purpose of assessing executive function and self-regulation. The BRIEF-A and other self-reported measures of executive functions have been useful in characterizing multidimensional behavioral difficulties in a wide variety of developmental (e.g., ADHD) and neurodegenerative conditions (e.g., fronto-temporal dementia and Alzheimer’s disease) [115,116]. The last assessment planned for each participant will be the Mini-Mental State Exam (MMSE), which is a widely used test of general cognitive function among the elderly and has recently been used to assess adult CHD patients [117]. It is also known as the Folstein Test or the Standardized Mini-Mental State Examination (SMMSE). This cognitive screening instrument assesses orientation, attention, memory, language, and visual–spatial skills without the need for special training requirements to administer it. The expected time for the execution of these three tasks should be no longer than 30 min, with the option for participants to complete the questionnaire remotely prior to their MRI scan.

We will begin with each metric from Specific Aims 1–3 that has a significant correlation with neurocognitive outcome. We will then search for the combination of factors (question 1 of Aim 4) from the questionnaire that best moderates the relationship by modeling CR as a latent variable (Figure 2, top) that, when interacting with brain abnormalities, best predicts cognitive outcome. Alternatively, we will use several machine learning techniques [106], focusing on naïve Bayes, ensemble learning methods (such as a random forest), and more sophisticated nonlinear approaches, including neural networks. These methods will be utilized to test the predictive accuracy of our models using 10-fold cross-validation. Statistical significance will be ascertained by the F1 score, Cohen’s kappa, and FDR-corrected at q < 0.05. Age and sex will be entered as covariates. Additionally, we will model three-way interactions (e.g., burden-X-age-X-CR) on the outcome, as the effect may vary with age [118] or sex [119], as has been seen in dementia populations. From the factor loadings and their standard errors, we will investigate whether the weightings are different according to the type of brain abnormality (injury, structure, physiology).

Secondary Analysis. As a preliminary examination of neurophysiological correlates (question 2 above), we will correlate our metric for CR with the neuroimaging data obtained in aims 2 and 3 using the methodology detailed in those aims (Figure 2, middle). Specifically, we will investigate both structural network topology (obtained from diffusion imaging), physiology (CVR) in other regions of the brain than found in Aim 3, and functional network topology (obtained from BOLD imaging) using metrics obtained from graph theory [120,121]. Functional topology has been previously shown to correlate with CR in elderly populations [122], and we have preliminary data that functional topology is abnormal in pre-adolescent CHD (Figure 2). Significant findings will provide evidence that specific structural, physiological, or functional differences are neurophysiological correlates of CR. It is of further interest to know whether these correlates are, in fact, a result of differing brain plasticity [123], as is hypothesized in theoretical models of CR [124]. Such models involve differential recruitment of cortical networks. To investigate this, we will use a mediation (indirect effects) model (Figure 2, bottom) to test whether our CR correlate is a suppressor (e.g., inverse mediation) of the relationship between pathology and outcome (e.g., the indirect pathway results in a better outcome). We will use bootstrap methods [125] to test the mediation results for statistical significance using bias-corrected and accelerated confidence intervals [126,127]. We have previously used mediation methods in an investigation of the relation between structural topology, neurocognitive outcome, and CHD adolescents with transposition of the great arteries [72].

### 2.9. Missing Data

See Supplemental Methods.

### 2.10. Multi-Center MRI Quality Assurance and Quality Control (QA/QC)

See Supplemental Methods and Supplemental Figures S4 and S5.

### 2.11. Data Transfer—See Supplemental Methods

Our study design has anticipated potential limitations to this study that may arise, as well as ways to mitigate these limitations:

Recruitment and Participant Fatigue: We have proposed recruitment from 13 sites, with small targeted recruitment for the neuroimaging ancillary study relative to the targeted enrollment of the parent MINDS study (n = 156 of 500). The parent study is one hour long, and we have multiple options for the patient to be scheduled for the brain MRI/questionnaire (90 min), so participant fatigue is less likely to happen.

Implantable Cardiac Devices: Due to our focus on individuals with ACHD, we expect all participants will have had corrective surgery at some point, with the majority having implanted hardware. These implants can range from occluders, closure devices, coils, stents, or valve replacements (commonly referred to as “passive implants” as they do not require an external power source) to active implanted medical devices such as an ICD or pacemaker (those requiring external power to operate). Because our primary goal is to scan participants at 3 T, extra MR safety precautions must be taken. All participants with an ICD, pacemaker, or retained pacing wires will be immediately excluded from participating in ancillary imaging due to these devices being ruled MR Unsafe at all field strengths. But those with coils, stents, and valve replacements will need to be identified and cleared prior to scanning at 3 T. Only devices determined to be MR Safe may proceed with enrollment. If a device is determined to be MR Conditional, further vetting will be needed as these devices are only imaging-compatible in specific operating conditions. Most devices manufactured before 2010 are “legacy implants” and may not carry an MR safety label. Those devices implanted before 1996 were installed before MRI compatibility was relevant and may not have been backward-tested by the manufacturer for compatibility at 3 T, such as the Palmaz 308 aortic stent. Even with this understanding, we do not expect many participants to have implants deemed unacceptable to scan above 1.5 T. But, with the possibility of there being exclusions for scanning at 3 T, a protocol can be eventually developed at 1.5 T (or lower Tesla) to allow for imaging at a lower field strength, providing data for gross brain metrics and injury.

Harmonization limitations during the COVID-19 pandemic and Alternative Harmonization Approach. For our QA plan and because of limitations for traveling during the COVID-19 pandemic, we performed initial QA using two phantoms that reside at each of the sites (ACR-anatomic and f-BIRN-functional) and two specific phantoms that are shipped to the sites (HARDI-diffusion—Supplemental Figure S4—and ASL-perfusion—Supplemental Figure S5) for QA purposes. In parallel, we have been leveraging prior data from five human phantoms who previously traveled to each PHN site approximately every six months. The QA/QC data will be used to establish the compatibility of data from different sites, the long-term reproducibility of the results at each site, and the development of a site-specific plan.

For retrospective harmonization achieved through analysis, we have previously controlled for the effect of the scanner/vendor on the neuroimaging metric/biomarker. This was accomplished by not only including the scanner/vendor as a covariate but also modeling different between-subject variances dependent on the scanner. It is noteworthy that we have demonstrated these variances to be similar for specific neuroimaging metrics [74]. However, this retrospective statistical harmonization approach does depend on a robust sample size per site, which may be an issue with our study as the sample size/site may be small. To overcome this potential limitation, we can adapt other statistical approaches, including functional normalization, RAVEL, surrogate variable analysis, or COMBAT, a popular batch adjustment method originally developed for genomics data [128,129,130]. COMBAT has recently been shown to be effective at reducing inter-scanner variation while preserving biological variation. COMBAT can also be effective with small sample size and provide regional-specific correction factors for multimodal neuroimaging data [128,129,130]. We have recently successfully applied this COMBAT technique to a four-center DTI dataset of n = 763 neonates with CHD [131].

## 3. Discussion

With advances in surgical, medical, and catheter-based interventions, most CHD patients are surviving into adulthood [2]. In recognition of the importance of this dramatic demographic shift, the NHLBI-funded PHN has expanded its research focus to encompass ACHD patients. Children and adolescents with CHD have well-documented deficiencies in multiple neurocognitive domains, including attention, executive functioning, language, and memory [59,60,61,132,133,134,135,136,137,138,139]. However, there is a paucity of correlative neurocognitive and neuroimaging studies in ACHD patients, a major knowledge gap addressed by this ancillary study. Impaired neurocognitive outcomes impact well-being and psychosocial morbidities, including social difficulty, lower education attainment, and greater unemployment. However, characterizing neurocognitive deficits is only the first step towards developing targeted management and treatment strategies to improve outcomes in ACHD patients. A detailed understanding of the brain correlates of such deficits and their interactions with lifestyle and behavioral factors is crucial.

The initial aims of our project are to understand the neural underpinnings of neurocognitive outcomes in ACHD patients (Aims 1–3). Some patients present signs of acquired vascular injury, but a substantial number of neurocognitively impaired CHD patients do not. This indicates more subtle etiologies of outcome, such as microstructural, neuronal, or vascular abnormalities. Indeed, risk factors throughout the developmental period are heterogeneous and include genetic variants, brain dysmaturation, postnatally acquired hypoxia/ischemia, and vascular abnormalities. Similar adverse neurocognitive outcomes may arise from a variety of different brain abnormalities (i.e., equifinality), yet optimal prevention and intervention strategies may differ depending upon the mechanism of brain injury. Our work in pediatric CHD patients to date has shown that white matter structural topology, cerebral blood flow (CBF), and cerebrovascular reserve (CR) are predictive of neurocognitive outcomes [80,138,139]. The MINDS–ACHD ancillary project will allow us to extend our research to an adult population to better understand the underpinnings of adverse neurodevelopmental outcomes in ACHD patients. We are pursuing a multimodal approach with MRI to detect acquired vascular-related brain injury and investigate subtler structural and vascular deficits, including cortical thickness, structural connectivity, and cerebrovascular reserve. These results will allow us to classify and stratify ACHD patients not only according to neurocognitive outcomes but also according to brain injury and structural and physiological characteristics.

The second major aim of our project is to elucidate the possible mechanisms of diminished cognitive reserve (CR) in the ACHD population. CR is a protective factor that prevents or reduces cognitive impairment in response to a given brain insult. CR is known to correlate with lifestyle and cognitive factors, such as level of education, type of occupation, vocabulary, and frequency of reading, which are frequently referred to as “cognitive enrichment” or “intellectual enrichment” factors [46,47,48,49]. While the physiological mechanism is still being investigated, theoretical models of CR and some clinical research studies are converging towards a correlation of CR with brain plasticity—the capacity of the brain to reorganize after insult. We hypothesize that a similar mechanism is at work in ACHD patients. We will derive a suitable proxy for CR using both moderation/mediation statistical models and machine learning techniques as the optimal way to estimate CR, which is currently unknown in this population. Obtaining a validated proxy for CR is essential for the design of optimal behavioral interventions, as studies in the elderly have shown that CR is modifiable over time and results in improved outcomes.

## 4. Conclusions

Our study, which utilizes advanced accelerated multimodal neuroimaging acquisition paired with rigorous and novel harmonization/post-processing tools, will identify structural–physiological correlates of neurocognitive outcomes, representing the first multi-center neuroimaging study to be performed in ACHD. Our main objective is to measure neuroimaging biomarkers (brain injury, structure, and physiology) and relate them to neurocognitive outcomes leveraged from the larger parent PHN MINDS–ACHD study. Importantly, other behavioral and environmental enrichment data will be integrated with these neuroimaging and neurocognitive outcome data to model CR. Our findings will inform the design of future longitudinal and/or interventional studies to improve neurocognitive outcomes and ultimately reduce cognitive decline in ACHD. Inferences from this research will help shape the care of ACHD patients and further our understanding of the interplay between brain injury and cognitive reserve.

## Data Availability

No new data were created or analyzed in this protocol/methods manuscript. Data sharing is not applicable to this article.

## Supplementary Materials

The following supporting information can be downloaded at: https://www.mdpi.com/article/10.3390/jcdd10090381/s1, Figure S1: Pipeline for Regional Cerebral Volumes; Figure S2: Diffusion imaging with multi-shell technique with multiple steps; Figure S3: Processing pipeline for ASL (cerebral blood flow) and BOLD (functional connectivity) neuroimaging data; Figure S4: High Angular Diffusion Imaging (HARDI) Phantom; Figure S5: Arterial Spin Label (ASL) Phantom; Table S1A: MR Harmonized Pulse Sequences proposed for the MINDS Neuroimaging Ancillary Studies for three MRI vendors; Table S1B: MR Harmonized Pulse Sequences for ASL (arterial spin labeling ) or Cerebral Blood Flow Measures proposed for the MINDS Neuroimaging Ancillary Studies for three MRI vendors.
