# Supplementary material for: Design and Harmonization Approach for the Multi-Institutional Neurocognitive Discovery Study (MINDS) of Adult Congenital Heart Disease (ACHD) Neuroimaging Ancillary Study: A Technical Note
Source: J Cardiovasc Dev Dis. 2023 Sep 6;10(9):381. doi: 10.3390/jcdd10090381 (PMC10532244; doi:10.3390/jcdd10090381)
Supplement: Supplementary file 1 [file jcdd-10-00381-s001.zip › jcdd-2539900-supplementary.pdf]

Supplemental Material

Design and Harmonization Approach for the Multi-Institutional Neurocognitive Discovery Study (MINDS) of Adult Congenital Heart Disease (ACHD) Neuroimaging Ancillary Study: A Technical Note

Supplemental Methods

Specific Aim 1: Vascular-Related Brain Injury – Is it associated with neurocognitive deficits? Methodology. Qualitative assessment of Acquired Brain Injury: We will perform qualitative assessment of acquired brain injury using the scoring developed using? a prior adolescent brain imaging cohort. Quantitative assessment of Acquired Brain Injury: 3D FLAIR images will be registered to the 3D T1-weighted images by using statistical parametric mapping software (SPM12, Wellcome Institute of Neurology, University College London, UK, http://www.fil.ion.ucl.ac.uk/spm/doc/), running on Matlab R2022a (Mathworks, Natick, MA, USA). WMH segmentations will be performed on the FLAIR scans by the lesion prediction algorithm as implemented in the Lesion Segmentation Toolbox version 2.0.15 (www.statisticalmodeling.de/lst.html) for SPM12.[1] WMH segmentations will be thresholded on a 0.5 probability, and WMH and cortical infarct volumes will be calculated using the Lesion Segmentation Toolbox. Hemosiderin lesions will be counted on the susceptibility-weighted imaging (SWI).

Specific Aim 2: Brain Structure – Is it associated with neurocognitive deficits?

Methodology: Cortical Thickness Measurement. We will use FreeSurfer for segmentation (Supplemental Figure 1) of cortical thickness and hippocampus as previously described by our group and others.[2,3] We have previously applied these methods to the neonatal and pediatric brain.[4,5] DTI Pre-processing (Supplemental Figure 2): All images will be corrected for motion, eddy current, and slice dropout artifacts using standard routines in FSL (FMRIB, Oxford UK). Standard DTI: DTI metrics, including fractional anisotropy (FA), and direction of the principal eigenvector will be computed for each voxel. Deterministic tractography will be performed using in-

house software written in IDL (http://www.ittvis.com, Boulder, CO). Streamlines will be computed from each white matter voxel (determined as all voxels with FA > 0.25) in both directions. Stopping thresholds for the tractography will be turning angle > 45 degrees or FA < 0.25. MB (multi-band)-DTI: For the MB-DTI data, due to the large number of directions, the orientation distribution function (ODF) will be computed according to routines in DSI Studio.[6-8] Tractography will be performed according to routines in DSI Studio. The ODF allows for the detection of crossing fiber tracts within a voxel and thus allows a more accurate reconstruction of fiber tracts than is possible with standard DTI. A more accurate metric of anisotropy generalized fractional anisotropy (GFA) will be computed. Streamlines will be computed from each white matter voxel (GFA > 0.25) and stopping thresholds will be turning angle > 45 degrees or GFA < 0.25.

Specific Aim 3: Brain Physiology – Is it associated with neurocognitive deficits? Methodology. ASL Pre-processing (Supplemental Figure 3). After motion correction, CBF maps will be computed using the two-compartment model[9,10] with literature values[11] used for arterial T1 and T1 of gray matter and arterial and tissue transit times and normalized into standardized (MNI) space . BOLD Pre-processing. We will closely follow published methods[12,13] in order to minimize the risk of spurious correlations due to participant motion. The process will include slice timing correction, motion correction, spatial normalization, volume censoring of motion-corrupted frames, regression out of nuisance (including motion correction) parameters and band-pass filtering. FCS in gray matter is defined as the average of the Pearson correlations between its voxel time course and all other voxel time courses in gray matter, with negative correlations being set to zero.[14] rCVR Maps. rCVR will be computed on a voxelwise basis as the negative of the ratio of FCS to CBF. To further enhance the robustness of this estimator, the CBF maps will be spatially filtered with a 3-X-3-X-3

boxcar filter (only including voxels in gray matter) prior to computation. The final value of rCVR will be the average of the two values computed from each of the BOLD runs. CVRe Maps. The $P_{ET}CO2$ will be estimated from the global BOLD signal via band-pass filtering as shown in,[15] and then each voxel time course will be correlated with $P_{ET}CO2$.

Missing Data: We anticipate minimal missing data with respect to the neuroimaging variables and, based on the knowledge of the completeness of the PHN MINDS datasets, minimal missing data for the participant/medical variables. Because these data are likely to be missing at random, simple mean imputation may be implemented if missing data are less than 5%, preserving mean unbiased estimates with minimal effect on associated standard errors. It is possible that neurocognitive assessments may be incomplete or out of allowable scoring windows for the instrument. We will conduct a comparison of key characteristics of the MINDS cohort members who do and do not have available or valid neurocognitive test scores, to assess the generalizability of our results to the broader young ACHD population.

Multicenter MRI Quality Assurance and Quality Control (QA/QC): Proper QA/QC procedures include a complex multi-step harmonization process which involves (1) development and surveillance of a standardized neuroimaging protocol across sites; (2) prospective on-going synthetic and human phantom studies to provide cross-calibration across scanners and; (3) retrospective or statistical harmonization of collected human subject neuroimaging data [knowing that inter-scanner variation will still occur despite implementation of (1) and (2)]. We will leverage our on-going rigorous harmonization approach for the Pediatric Heart Network SVRIII Brain Connectome study (ClinicalTrials.gov Registration Number: NCT02692443) which incorporates a QA

procedure[16] used by multiple NIH-funded multi centered studies including the Human Brain Connectome, TRACK-TBI and the Pediatric Brain Tumor Consortium.[17-26] For the MINDS neuroimaging ancillary study, we will use predominately both Siemens Prisma and Philips Achieva 3T MRI systems. We currently have a database of greater than 75 synthetic and human phantom HARDI diffusion tensor data on Pediatric Heart Network 3T MRI scanners (Siemens /Phillips vendors). We have recently developed a new pipeline that incorporates both synthetic phantom and human individual subject-specific template/tract generation via a semi-automated approach. We can leverage this ongoing database to perform PHN inter-site reliability studies to provide assurance that neuroimaging studies being performed on the proposed MINDS scanners can be compared.[27,28] We will assess diffusion QA data using the HARDI Phantoms (Supplemental Figure 4).[28-30] For the DTI phantom and human studies, we will evaluate multiple values: (1) SNR at the center and periphery of the phantom; (2) comparison of image distortion in phase-encoding direction between EPI and spin echo image; and (3) estimation of FA and mean diffusivity (MD) measured by region-of-interest (ROI) analysis. For resting state data and the fBIRN phantom we will use Weisskoff plots and guidelines of fBIRN research group including average tSNR.[31-35] Anatomic quality (T1/T2 weighted) will be done according to American College of Radiology (ACR) guidelines[16] and will include geometry accuracy, high contrast spatial resolution, the accuracy of slice thickness and position, image intensity homogeneity, and low-contrast object detectability. We will ensure harmonization of the ASL pulse sequence between PHN/MINDS Siemens and Philips 3T scanners. Our team has experience with pulse sequence design for ASL across different vendor platforms.[11,36] We will be using recently published standards for ASL perfusion acquisition and quantitation.[37] QASPER is an advanced calibration phantom for ASL. Its features include a unique MRI compatible pump design, the use of proprietary technology to

simulate capillary blood flow and is calibrated to international standards (Supplemental Figure 5).

Data Transfer: Imaging data transfer and subject forms from outside institutions will be uploaded through a secure, SSL encrypted portal hosted at the University of Pittsburgh, with individual user authentication and institution-restricted access. The upload portal runs on the latest version of XNAT, an open-source informatics platform developed for the high throughput management and sharing of imaging data.[18,38-40]

SUPPLEMENTAL METHODS REFERENCES:

1. Kant, I.M., *et al.* The association between brain volume, cortical brain infarcts, and physical frailty. *Neurobiology of aging* **70**, 247-253 (2018).
2. Shi, J., *et al.* Genetic influence of apolipoprotein E4 genotype on hippocampal morphometry: An N= 725 surface-based Alzheimer's disease neuroimaging initiative study. *Human brain mapping* **35**, 3903-3918 (2014).
3. Shi, J., Thompson, P.M., Gutman, B., Wang, Y. & Initiative, A.s.D.N. Surface fluid registration of conformal representation: Application to detect disease burden and genetic influence on hippocampus. *NeuroImage* **78**, 111-134 (2013).
4. Lao, Y., *et al.* Thalamic alterations in preterm neonates and their relation to ventral striatum disturbances revealed by a combined shape and pose analysis. *Brain Structure and Function*, 1-20 (2014).
5. Shi, J., *et al.* A multivariate surface-based analysis of the putamen in premature newborns: regional differences within the ventral striatum. *PloS one* **8**, e66736 (2013).
6. Malykhin, N., Concha, L., Seres, P., Beaulieu, C. & Coupland, N.J. Diffusion tensor imaging tractography and reliability analysis for limbic and paralimbic white matter tracts. *Psychiatry Research: Neuroimaging* **164**, 132-142 (2008).
7. Jiang, H., van Zijl, P., Kim, J., Pearlson, G.D. & Mori, S. DtiStudio: resource program for diffusion tensor computation and fiber bundle tracking. *Computer methods and programs in biomedicine* **81**, 106-116 (2006).
8. Yeh, F.-C., Wedeen, V.J. & Tseng, W.-Y.I. Estimation of fiber orientation and spin density distribution by diffusion deconvolution. *NeuroImage* **55**, 1054-1062 (2011).
9. Alsop, D.C. & Detre, J.A. Reduced transit-time sensitivity in noninvasive magnetic resonance imaging of human cerebral blood flow. *Journal of cerebral blood flow and metabolism : official journal of the International Society of Cerebral Blood Flow and Metabolism* **16**, 1236-1249 (1996).
10. Wang, J., *et al.* Comparison of quantitative perfusion imaging using arterial spin labeling at 1.5 and 4.0 Tesla. *Magnetic resonance in medicine* **48**, 242-254 (2002).
11. Schmithorst, V.J., *et al.* Optimized simultaneous ASL and BOLD functional imaging of the whole brain. *Journal of magnetic resonance imaging : JMRI* **39**, 1104-1117 (2014).
12. Power, J.D., *et al.* Methods to detect, characterize, and remove motion artifact in resting state fMRI. *NeuroImage* **84**, 320-341 (2014).
13. Power, J.D., Schlaggar, B.L. & Petersen, S.E. Recent progress and outstanding issues in motion correction in resting state fMRI. *NeuroImage* **105**, 536-551 (2015).
14. Liang, X., Zou, Q., He, Y. & Yang, Y. *Coupling of functional connectivity and regional cerebral blood flow reveals a physiological basis for network hubs of the human brain*, (2013).
15. Liu, P., *et al.* Cerebrovascular reactivity mapping without gas challenges. *NeuroImage* **146**, 320-326 (2017).
16. Mulkern, R.V., *et al.* Establishment and Results of a Magnetic Resonance Quality Assurance Program for the Pediatric Brain Tumor Consortium. *Acad Radiol* **15**, 1099-1110 (2008).
17. Poussaint, T.Y., *et al.* The Neuroimaging Center of the Pediatric Brain Tumor Consortium-collaborative neuroimaging in pediatric brain tumor research: a work in progress. *AJNR Am J Neuroradiol* **28**, 603-607 (2007).

18. Marcus, D.S., *et al.* Human Connectome Project informatics: quality control, database services, and data visualization. *NeuroImage* **80**, 202-219 (2013).
19. Elam, J.S. & Van Essen, D. Human Connectome Project. in *Encyclopedia of Computational Neuroscience* 1-4 (Springer, 2014).20. Sotiropoulos, S.N., *et al.* Advances in diffusion MRI acquisition and processing in the Human Connectome Project. *Neuroimage* **80**, 125-143 (2013).
21. Van Essen, D.C., *et al.* The WU-Minn human connectome project: an overview. *NeuroImage* **80**, 62-79 (2013).
22. Zuo, X.-N. & Xing, X.-X. Test-retest reliabilities of resting-state FMRI measurements in human brain functional connectomics: a systems neuroscience perspective. *Neuroscience & Biobehavioral Reviews* (2014).
23. Dams-O'Connor, K., *et al.* The Impact of Previous Traumatic Brain Injury on Health and Functioning: A TRACK-TBI Study. *Journal of neurotrauma* **30**, 2014-2020 (2013).
24. Yue, J.K., *et al.* Transforming research and clinical knowledge in traumatic brain injury pilot: multicenter implementation of the common data elements for traumatic brain injury. *Journal of neurotrauma* **30**, 1831-1844 (2013).
25. Yuh, E.L., *et al.* Diffusion Tensor Imaging for Outcome Prediction in Mild Traumatic Brain Injury: A TRACK-TBI Study. *Journal of neurotrauma* (2014).
26. Owen, J.P., *et al.* Test–Retest Reliability of Computational Network Measurements Derived from the Structural Connectome of the Human Brain. *Brain connectivity* **3**, 160-176 (2013).
27. Walker, L., Curry, M., Nayak, A., Lange, N. & Pierpaoli, C. A framework for the analysis of phantom data in multicenter diffusion tensor imaging studies. *Human brain mapping* **34**, 2439-2454 (2013).
28. Keenan, K. Quantitative Magnetic Resonance Imaging and Phantom Development. *Bulletin of the American Physical Society* **59**(2014).
29. Selwyn, R. Phantoms for Magnetic Resonance Imaging. in *The Phantoms of Medical and Health Physics* 181-199 (Springer, 2014).
30. Vannier, M.W. Traumatic Brain Injury Diffusion Magnetic Resonance Imaging Research Roadmap Development Project. (DTIC Document, 2010).
31. Glover, G.H., *et al.* Function biomedical informatics research network recommendations for prospective multicenter functional MRI studies. *Journal of Magnetic Resonance Imaging* **36**, 39-54 (2012).
32. Friedman, L. & Glover, G.H. Report on a multicenter fMRI quality assurance protocol. *Journal of Magnetic Resonance Imaging* **23**, 827-839 (2006).
33. Friedman, L., Glover, G.H. & Consortium, F. Reducing interscanner variability of activation in a multicenter fMRI study: controlling for signal-to-fluctuation-noise-ratio (SFNR) differences. *Neuroimage* **33**, 471-481 (2006).
34. Friedman, L., Glover, G.H., Krenz, D. & Magnotta, V. Reducing inter-scanner variability of activation in a multicenter fMRI study: role of smoothness equalization. *Neuroimage* **32**, 1656-1668 (2006).
35. Friedman, L., *et al.* Test–retest and between-site reliability in a multicenter fMRI study. *Human brain mapping* **29**, 958-972 (2008).
36. Schmithorst, V.J., *et al.* Evidence that neurovascular coupling underlying the BOLD effect increases with age during childhood. *Human brain mapping* **36**, 1-15 (2015).

37. Alsop, D.C., *et al.* Recommended implementation of arterial spin-labeled perfusion MRI for clinical applications: A consensus of the ISMRM perfusion study group and the

European consortium for ASL in dementia. *Magnetic resonance in medicine* **73**, 102-116 (2015).
38. Keator, D.B.*, et al.* A national human neuroimaging collaboratory enabled by the Biomedical Informatics Research Network (BIRN). *Information Technology in Biomedicine, IEEE Transactions on* **12**, 162-172 (2008).
39. Marcus, D.S.*, et al.* Informatics and data mining tools and strategies for the human connectome project. *Frontiers in neuroinformatics* **5**(2011).
40. Marcus, D.S., Olsen, T.R., Ramaratnam, M. & Buckner, R.L. The extensible neuroimaging archive toolkit. *Neuroinformatics* **5**, 11-33 (2007).

Supplemental Figures and Figure Legend:

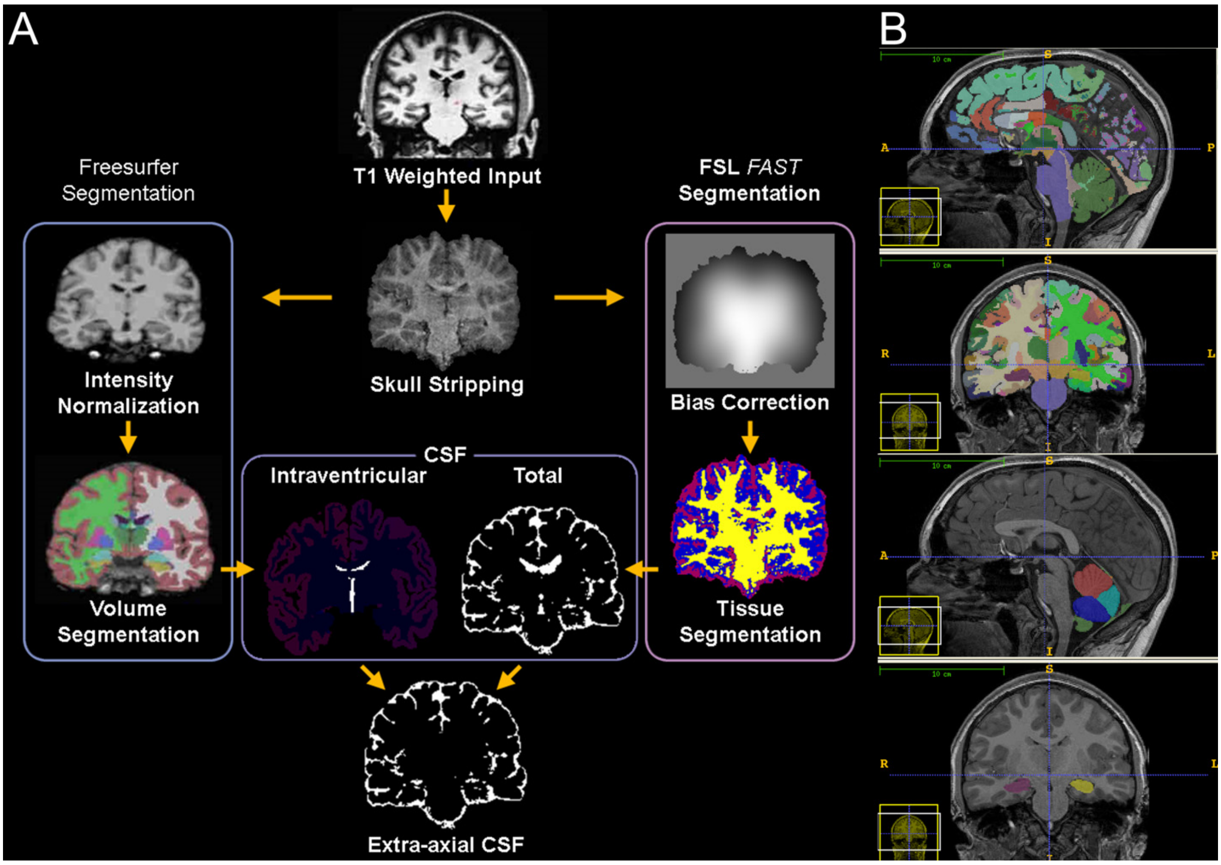

Supplemental Figure S1: Pipeline for Regional Cerebral Volumes: Volumetric segmentation of regional supratentorial and infratentorial cerebral volumes with manual correction of selected focus regions including subdivisions of the cerebellum and the hippocampus. Specialized pipelines for quantification of different CSF (cerebral spinal fluid) are noted.

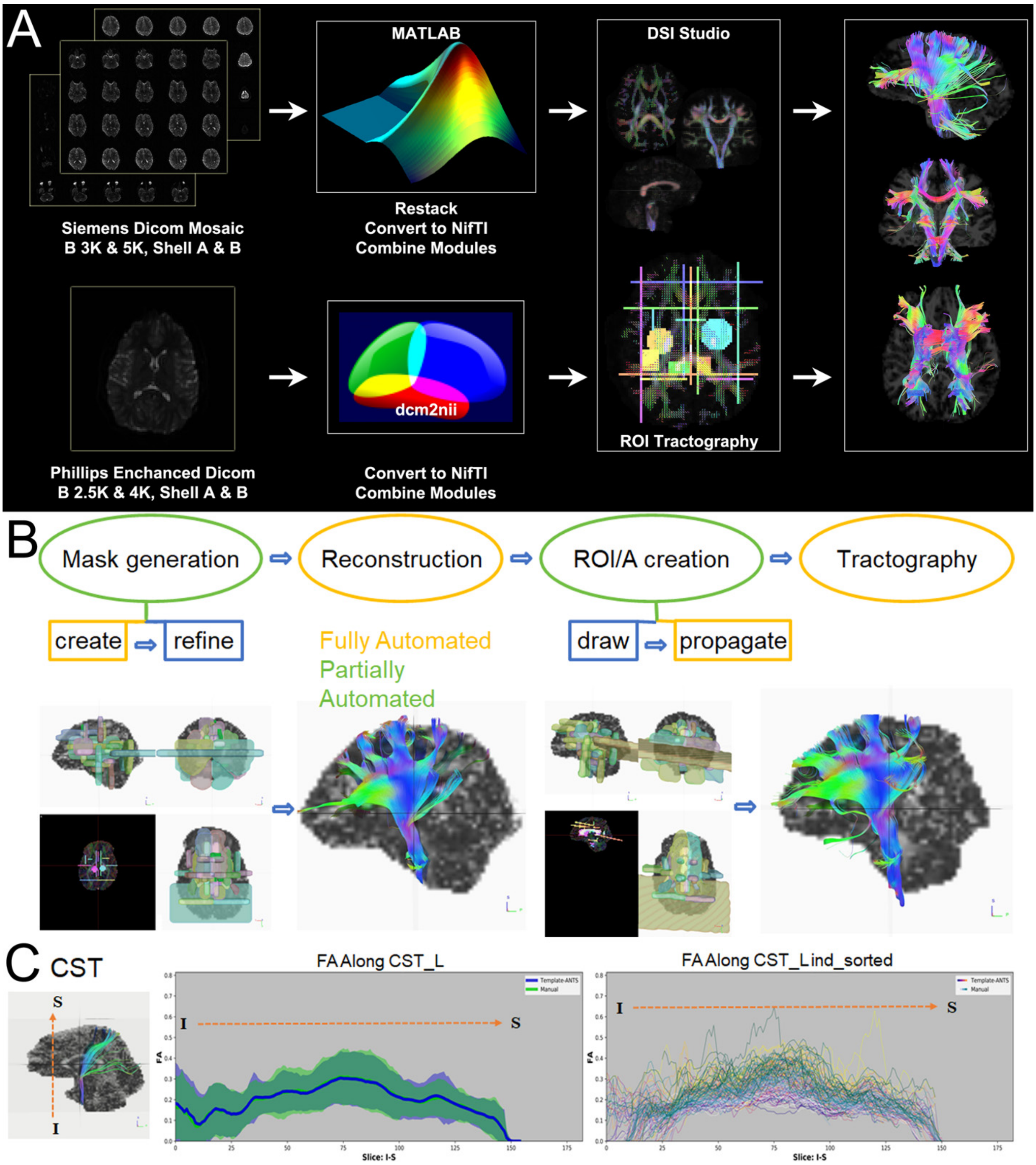
A
MATLAB
DSI Studio
Siemens Dicom Mosaic
B 3K & 5K, Shell A & B
Restack
Convert to NifTI
Combine Modules
dcm2nii
ROI Tractography
Phillips Enchanced Dicom
B 2.5K & 4K, Shell A & B
Convert to NifTI
Combine Modules
B
Mask generation
Reconstruction
ROI/A creation
Tractography
create
refine
Fully Automated
Partially
Automated
draw
propagate
C CST
FA Along CST_L
FA Along CST_Lind_sorted
Template-ANTS
Manual
Slice: I-S
FA

Supplemental Figure S2: Diffusion imaging with multi-shell technique with multiple steps (Mask Generation, Reconstruction, Region of Interest/Avoidance and generation of tractography) for creating specific cerebral white matter tracts; Bottom row shows generation of along tract white matter plots that show variation of diffusion metrics (including fractional anisotropy) in relation to anatomic locations.

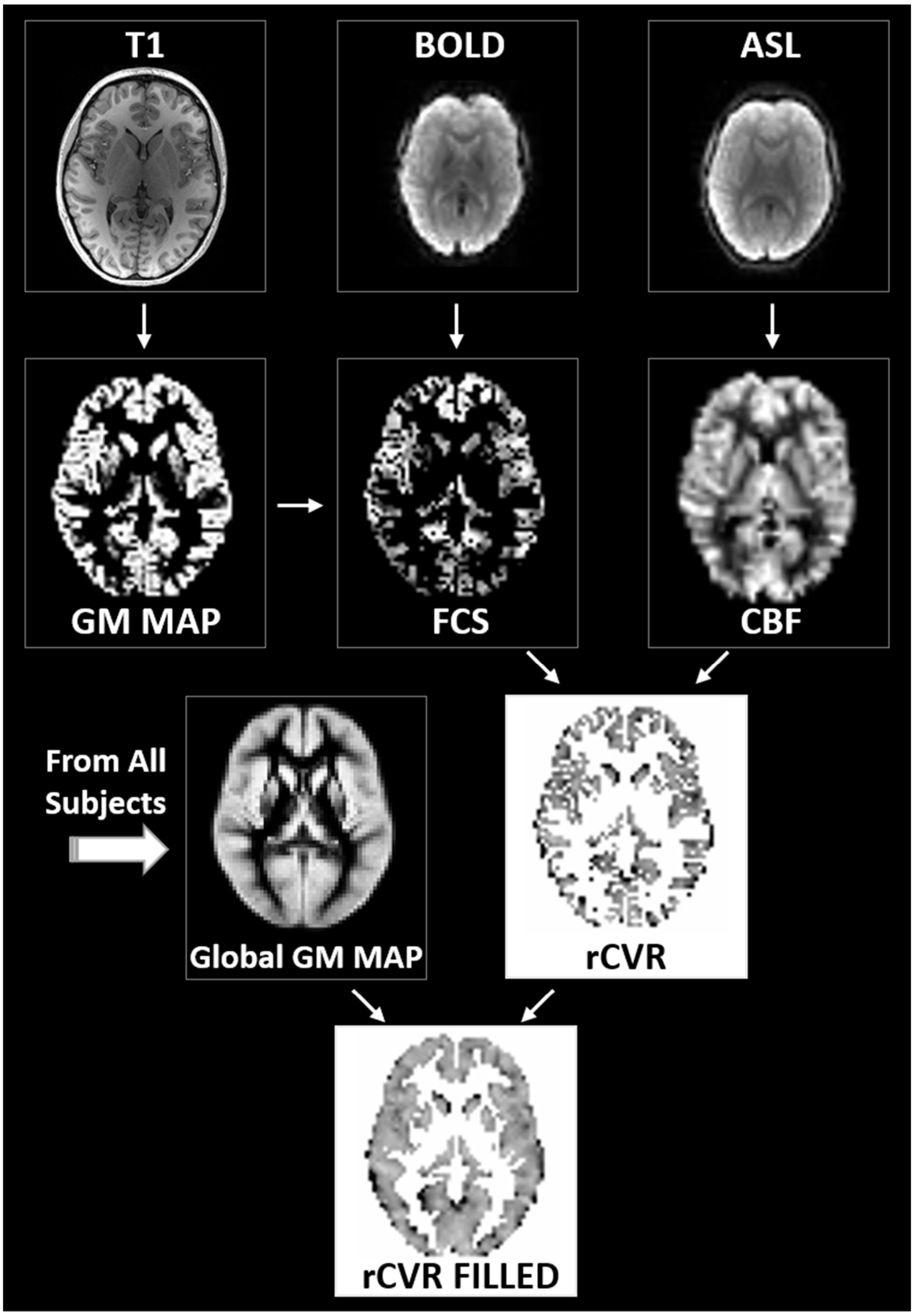

Supplemental Figure S3: Processing pipeline for ASL (cerebral blood flow) and BOLD (functional connectivity) neuroimaging data.

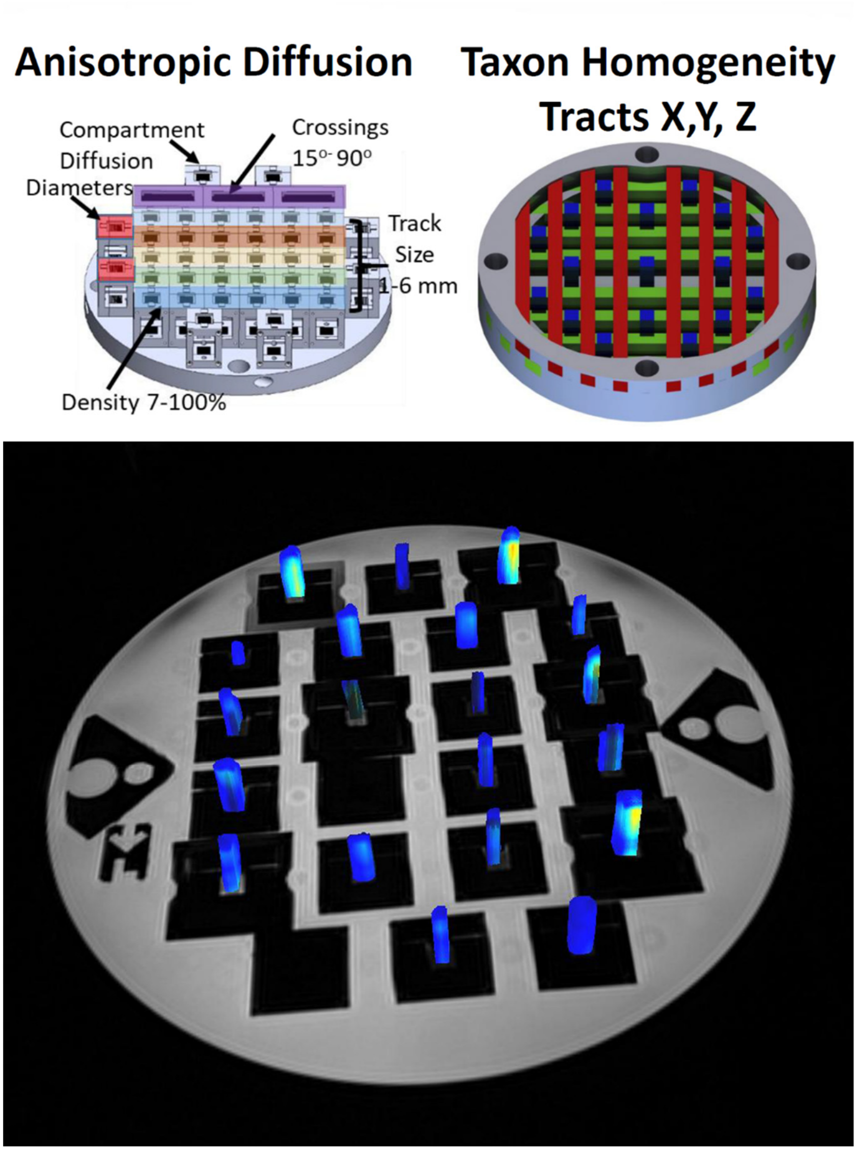

Supplemental Figure S4. High Angular Diffusion Imaging (HARDI) Phantom. The artificial axons made from textile fibers (Taxon) are arranged in standardized blocks of graduated density and bundle size onto a fully modular disc (top left). The taxons provide direction of anisotropy and a calibrated measurement for that anisotropy as FA, AD, RD, and MD values (top right). The HARDI images are reconstructed, and the taxon bundles are visualized as tracts (bottom).

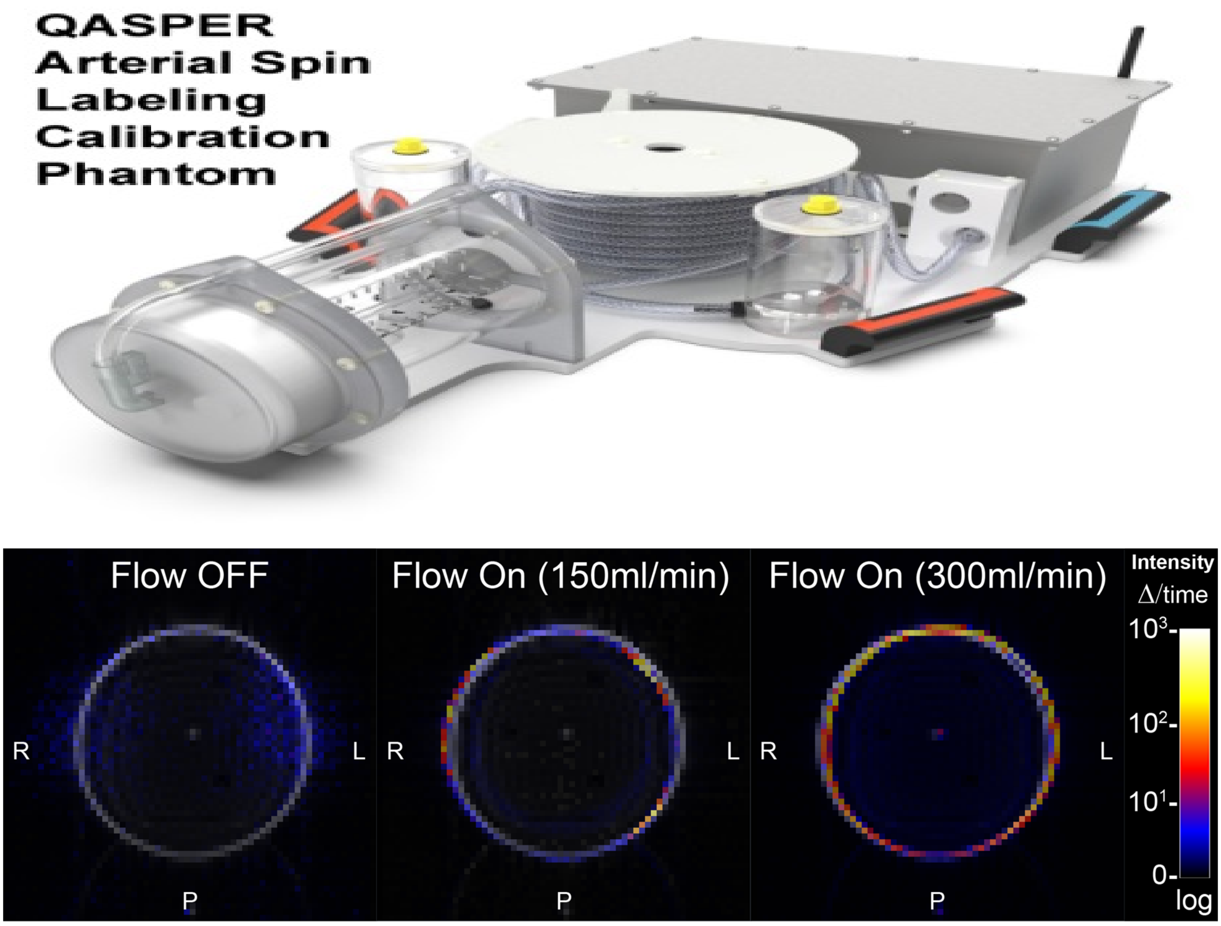

Supplemental Figure S5: Arterial Spin Label (ASL) Phantom: QASPER is an advanced calibration phantom for ASL. Its features include a unique MRI compatible pump design (top), the use of proprietary technology to simulate capillary blood flow and is calibrated to international standards (bottom).

Supplemental Table S1A: MR Harmonized Pulse Sequences proposed for the MINDS Neuroimaging Ancillary Studies for three MRI vendors

T1			**Matrix**	**slices**	**FOV**	**Resolution (mm)**	**TR (ms)**	**TE (ms)**	**TI (ms)**	**Flip Angle (deg)**	**Parallel Imaging**	**Multi-Band Acceleration**	**Phase partial Fourier**	**b-values**	**Acquisition Time**
	Siemens	T1	256 x 256	176	256 x 256	1.0 x 1.0 x 1.0	2500	2.88	1060	8	2x	Off	Off	N/A	7:12
	Philips	T1	256 x 256	225	256 x 240	1.0 x 1.0 x 1.0	6.31	2.9	1060	8	1.5 x 2.2	Off		N/A	5:38
	GE	T1	256 x 256	208	256 x 256	1.0 x 1.0 x 1.0	2500	2	1060	8	2x	Off	Off	N/A	6:09
SAG T2 FLAIR	Siemens		256x256	160	256x256	1.0x1.0x1.0	4800	120	1650	40	2x	Off	7/8	N/A	6:30
	Philips		256x256	160	256x256	1.0x1.0x1.2	4800	119	1650	40	N/A	Off	N/A	N/A	5:46
	GE		186x186	160	256x256	1.4x1.4x1.2	4800	119	1528	N/A	2x	Off	N/A	N/A	6:10
fMRI Resting Bold	Siemens	rs-fMRI	90 x 90	60	216 x 216	2.4 x 2.4 x 2.4	800	30	N/A	52	Off	6	Off	N/A	
	Philips	rs-fMRI	90 x 90	60	216 x 216	2.4 x 2.4 x 2.4	800	30	N/A	52	Off	6	0.9	N/A	
	GE	rs-fMRI	90 x 90	60	216 x 216	2.4 x 2.4 x 2.4	800	30	N/A	52	Off	6	Off	N/A	
Diffusion Imaging	Siemens	DTI Multiband	140 x 140	81	240 x 240	1.7 x 1.7 x 1.7	4100	88	N/A	90	Off	3	6/8	550 (6-dirs) 1000 (15-dirs) 2000 (15-dirs) 3000 (60-dirs)	7:31
	Philips	DTI Multiband	140 x 140	81	240 x 240	1.7 x 1.7 x 1.7	5300	89	N/A	78	Off	3	0.6	550 (6-dirs) 1000 (15-dirs) 2000 (15-dirs) 3000 (60-dirs)	9:14
	GE	DTI Multiband	140 x 140	81	240 x 240	1.7 x 1.7 x 1.7	4100	81.9	N/A	77	Off	3	5.5/8	550 (6-dirs) 1000 (15-dirs) 2000 (15-dirs) 3000 (60-dirs)	7:30
Axial SWI 3D	Siemens	256x230	80	210x192	0.8x0.8x2	27	20	N/A	15	2x	Off	Off	N/A	N/A	4:54
	Philips	384x315	75	230x190	0.6x0.6x2	31	13.4	N/A	17	N/A	Off	Off	N/A	N/A	3:36
	GE	256x232	80	210x192	0.8x0.8x2	36	25.2	N/A	15	N/A	Off	Off	N/A	N/A	4:50

Supplemental Table S1B: MR Harmonized Pulse Sequences for ASL (arterial spin labeling) or Cerebral Blood Flow Measures proposed for the MINDS Neuroimaging Ancillary Studies for three MRI vendors

Arterial Spin Labeling

		Matrix	slices	FOV	% FOV phase	Resolution (mm)	TR (ms)	TE (ms)	Flip Angle (deg)	Parallel Imaging	Multi-Band Acceleration	Phase partial Fourier	Label: Duration/ delay (ms)	Diffusions Directions	b-values	Acquisition Time
Siemens	PCASL	64	32	256	100	4.0x4.0x4.0	4000	13	90	2x	Off	Off	1500/1500	N/A	N/A	6:12
Philips	ASL	64	40	192	100	3.0x3.0x4.0	5000	16	40	Off	Off	Off	2000/2000	N/A	N/A	5:10
GE	ASL	64	32	240	100	4.0x4.0x4.0	N/A	N/A	N/A	Off	Off	Off	1525/1525	N/A	N/A	5:00
